# Flourishing among adolescents living with chronic pain and their parents: A scoping review

**DOI:** 10.1002/pne2.12088

**Published:** 2022-10-07

**Authors:** Ryan D. Parsons, Joanna L. McParland, Sarah L. Halligan, Liesbet Goubert, Abbie Jordan

**Affiliations:** ^1^ Department of Psychology University of Bath Bath UK; ^2^ Centre for Pain Research University of Bath Bath UK; ^3^ Department of Psychology Glasgow Caledonian University Glasgow UK; ^4^ Department of Psychiatry and Mental Health University of Cape Town Cape Town South Africa; ^5^ Department of Experimental‐Clinical and Health Psychology Ghent University Ghent Belgium

**Keywords:** adolescent, benefit finding, chronic pain, flourishing, positive psychology, social support

## Abstract

Evidence exists regarding the impact of flourishing in individuals living with chronic pain, but there are currently no reviews which collate the literature on flourishing in adolescents living with chronic pain and their parents. Therefore, the aim of this scoping review was to map and review the current literature, to document how flourishing is defined and understood in the literature, and to identify gaps in the field. Six databases were searched (Web of Science, Medline, Embase, APA PsycNet and the Cochrane Central Register of Controlled Trials). In addition, a limited gray literature search was conducted. The resulting data were collated and reported in relation to the review questions, by examining the included papers to search for the presence of flourishing. Database searches resulted in 7326 papers after duplicate removal, with eight remaining papers being assessed for full‐text eligibility. Following full‐text screening, a final four papers were included in the review. Within the papers, flourishing was defined in relation to commonalities of benefit finding, enhanced maturity and growth, and social support. Gaps in the literature and directions for future research are considered. This review suggests that there is a dearth of knowledge and research regarding flourishing among adolescents living with chronic pain and their parents, despite aspects of flourishing identified in limited literature. This warrants further investigation.

## 1 INTRODUCTION

Chronic pain (recurring or persistent pain lasting longer than 3 months^1^) is a common experience in childhood and beyond. A meta‐analysis by King et al.^2^ found that 11%–38% of young people report experiencing chronic pain, with rates of chronic pain typically increasing during adolescence. Such rates alongside a high number of adolescents (35%) reporting the experience of chronic pain into adulthood has identified adolescent chronic pain as a public health concern.^3^, ^4^

The impact of chronic pain on adolescent life is wide‐ranging and often deleterious, affecting familial, emotional, social, physical, school, and developmental functioning.^5^, ^6^, ^7^, ^8^, ^9^, ^10^, ^11^ However, despite a dominant focus on disability in the chronic pain literature, some evidence exists regarding flourishing while living with chronic pain.^8^ For example, a study by Trompetter et al.^12^ conducted with two adult chronic pain samples concluded that those living with chronic pain are able to flourish, while a qualitative study by Umberger and Risko^13^ observed positive growth in children and adolescents of parents living with chronic pain. The experiences of these children and adolescents led to growth in areas such as improved understanding and skills, and stronger character development and spirituality. It is likely that similar findings may be applicable to adolescents living with chronic pain and/or their parents.

There is currently no single agreed upon conceptualization of flourishing.^14^ In the context of mental health, Keyes^15^, ^16^ considers flourishing to be achieved when high levels of psychological, social, and emotional well‐being are experienced together, while Huppert and So^17^ conceptualize flourishing as “life going well.” Taking a slightly different approach, Goubert and Trompetter^8^ describe the construct of sustainability (used interchangeably with flourishing) as long‐term positive outcomes and optimal well‐being despite adversity. For the purposes of this review, we use flourishing as an umbrella term to describe positive changes in functioning and include related constructs such as benefit finding, hope, optimism, resilience, pain self‐efficacy, psychological flexibility, and acceptance,^8^, ^18^ or Post‐Traumatic Growth^19^ under this umbrella term. Therefore, our definition of flourishing extends beyond a simple reduction of negative outcomes and into the realm of positive change, either as a result of, or despite, chronic pain. However, despite our suggested definition, given the heterogeneity in how flourishing is recognized in the literature, within this review, we also include an investigation of how flourishing is defined and understood.

To fully understand flourishing in adolescents living with chronic pain, it is also important to acknowledge the wider social context in which they live. This includes the importance of considering the influence of parents and peers on adolescent chronic pain. A strong relationship with friends and adaptive parents may positively affect adolescent pain outcomes,^20^ while studies have found that higher family conflict and reduced adolescent autonomy are associated with increased depression and functional impairment,^21^, ^22^ which may also impact flourishing. Parents can serve as important role models in the context of pain^3^ and a key means of support can include strong family relationships.^23^ In addition to positive outcomes for adolescents due to strong family relationships, parenting an adolescent with chronic pain may also have a positive impact on parental well‐being by means of a bi‐directional relationship between adolescent and parent.^24^

To our knowledge, no reviews currently exist which report and synthesize the literature on flourishing in adolescents living with chronic pain and their parents. This collation may serve to broaden our understanding of the current literature and prove useful to inform future studies. Additionally, gaining a better understanding of flourishing in chronic pain could be useful to improve and expand on chronic pain interventions^12^ which promote growth and positive change over time.^13^ In particular, a positive focused approach could be especially useful to expand treatments in the context of a more typically deficits‐based approach to care for individuals living with chronic pain.

Therefore, the overall aim of this scoping review was to identify, map, and review the current literature, as well as identify gaps in the field.^25^ Using original methodological guidance provided by Arksey and O'Malley^26^ and updated guidance provided by Peters et al.^27^ for further consideration, we devised broad research questions which would enable us to generate appropriate breadth of coverage. Consequently, this review aimed to address the following research questions which considered flourishing in the context of adolescents who experience chronic pain and their parents:

1. What is the current scope of the literature with regard to flourishing in the context of adolescent chronic pain?
2. How is flourishing defined and understood in the pediatric pain literature?
3. What gaps exist in the current literature with regard to flourishing in the context of adolescent chronic pain and what are the directions for future research in the field?

## 2 LITERATURE SEARCH METHODS

### 2.1 Approach to the scoping review

This scoping review followed published methodological procedures as outlined in Arksey and O'Malley.^26^ The methodology includes a five‐stage process; (1) identifying the research questions, (2) identifying relevant studies, (3) study selection, (4) charting the data, and lastly (5) collating, summarizing, and reporting the results. Although not necessarily required for a scoping review,^26^ it is good practice to include a quality appraisal to facilitate a robust review.^28^ Therefore, the quality of the included studies was evaluated during data extraction using an appraisal tool.^29^ The protocol for this scoping review is registered with the Open Science Framework (OSF) database: https://osf.io/xrhnm/?view_only=49b4b1b4b5e44f7a83cada1e0edee784.

### 2.2 Search strategy

A comprehensive search strategy was developed in consultation with specialist subject librarians at both the University of Bath and Glasgow Caledonian University. Literature searches were conducted in July 2020 in five major databases by the first author (RP): Web of Science, Medline, Embase, APA PsycNet and the Cochrane Central Register of Controlled Trials. In addition, a limited gray literature search was conducted of conference abstracts as well as unpublished theses using EThOS. Although books were excluded, relevant book chapters were set aside for reference sections to be screened to augment the search. The search strategy involved searching for titles, abstracts, keywords, and MeSH terms as appropriate for each database, using a combination of four search strings. Search strings were developed following pilot searches in order to refine the search strategy and reduce irrelevant results, while retaining a broader focus. These search strings included *Age*, *Chronic Pain*, *Flourishing* and *Positive Associations* (see Table 1 for full list of search terms used). An example of a search strategy from APA PsycNet is shown in Table 2. Searches were limited to English only and no limits were placed on dates of publication.

**TABLE 1:** Search terms used under each search string

Age	Child* OR Adoles* OR “Late Childhood” OR “Young Adult” OR “Early Adulthood” OR “Young People” OR Young* OR Youth* OR “Young Person” OR Paediatric OR Pediatric OR Teen* OR Juveni* OR Minor* Or Pubesc*
Chronic pain	“Chronic Pain” OR Pain* OR “Long‐Term Pain” OR “Persistent Pain” OR “Recurrent Pain”
Flourishing	Flourish* OR “Positive Change” OR Grow* OR Thriv* OR Benefit OR Goal OR “Emotional Development” OR Well‐Being OR Well Being OR Adjustment OR Silver Lining OR Silver‐Lining OR Social OR Change OR Optimism OR Hope OR “Plans for Future” OR Self‐Efficacy OR Self Efficacy OR “Sense of Control” OR “Positive Affect” OR Sustain* OR Independence OR Identity OR “Developmentally Ahead” OR Acceptance OR Autonomy OR Character OR Spirit* OR “Treatment of Others” OR Engaged OR “Purpose in Life” OR Adjustment OR Optimism OR “Engagement in Meaningful Activities” OR “Quality of Life” OR “Fulfilling Life” OR Asset* OR “Mood” OR “Post Traumatic Growth” OR “Post‐Traumatic Growth” OR “Adversarial Growth” OR Satisf*
Positive associations	Flexib* OR Resilien* OR Positiv* OR Enhanc* OR Develop* OR Increas* OR Improv* OR “Better” OR Strong* OR “Enhanced Maturity” OR “Increased Well‐Being” OR “Improved Interpersonal Relationships” OR “Improved Relationship” OR “Better Adjustment” OR “Improved Social Relationships” OR “Increased Self‐Efficacy” OR “Increased Independence” OR “Improved Identity” OR “Increased Acceptance” OR “Increased Autonomy” OR “Stronger Character” OR “Nurtured Spirituality” OR “Engaged Living” OR “Better Adjustment” OR “Life Satisfaction” OR “Valued Activities” OR parent* OR Caregiver OR Care‐Giver

**TABLE 2:** APA PsycNet search strategy

((((**abstract**: [Child*]) *OR* (**abstract**: [Adoles*]) *OR* (**abstract**: [“Late Childhood”]) *OR* (**abstract**: [“Young Adult”]) *OR* (**abstract**: [“Early Adulthood”]) *OR* (**abstract**: [“Young People”]) *OR* (**abstract**: [Young*]) *OR*(**abstract**: [Youth*]) *OR* (**abstract**: [“Young Person”]) *OR* (**abstract**: [Paediatric]) *OR* (**abstract**: [Pediatric]) *OR* (**abstract**: [Teen*]) *OR* (**abstract**: [Juveni*]) *OR*(**abstract**: [Minor*]) *OR* (**abstract**: [Pubesc*]))) *OR* (((**Keywords**: [Child*]) *OR*(**Keywords**: [Adoles*]) *OR* (**Keywords**: [“Late Childhood”]) *OR* (**Keywords**: [“Young Adult”]) *OR* (**Keywords**: [“Early Adulthood”]) *OR* (**Keywords**: [“Young People”]) *OR* (**Keywords**: [Young*]) *OR* (**Keywords**: [Youth*]) *OR* (**Keywords**: [“Young Person”]) *OR* (**Keywords**: [Paediatric]) *OR* (**Keywords**: [Pediatric]) *OR*(**Keywords**: [Teen*]) *OR* (**Keywords**: [Juveni*]) *OR* (**Keywords**: [Minor*]) *OR*(**Keywords**: [Pubesc*])))) *AND* ((((**Keywords**: [Flexib*]) *OR* (**Keywords**: [Resilien*]) *OR* (**Keywords**: [Positiv*]) *OR* (**Keywords**: [Enhanc*]) *OR* (**Keywords**: [Develop*]) *OR* (**Keywords**: [Increas*]) *OR* (**Keywords**: [Improv*]) *OR* (**Keywords**: [“Better”]) *OR* (**Keywords**: [Strong*]) *OR* (**Keywords**: [“Enhanced Maturity”]) *OR*(**Keywords**: [“Increased Well‐Being”]) *OR* (**Keywords**: [“Improved Interpersonal Relationships”]) *OR* (**Keywords**: [“Improved Relationship”]) *OR* (**Keywords**: [“Better Adjustment”]) *OR* (**Keywords**: [“Improved Social Relationships”]) *OR*(**Keywords**: [“Increased Self‐Efficacy”]) *OR* (**Keywords**: [“Increased Independence”]) *OR* (**Keywords**: [“Improved Identity”]) *OR* (**Keywords**: [“Increased Acceptance”]) *OR* (**Keywords**: [“Increased Autonomy”]) *OR* (**Keywords**: [“Stronger Character”]) *OR* (**Keywords**: [“Nurtured Spirituality”]) *OR* (**Keywords**: [“Engaged Living”]) *OR* (**Keywords**: [“Better Adjustment”]) *OR* (**Keywords**: [“Life Satisfaction”]) *OR* (**Keywords**: [“Valued Activities”]) *OR* (**Keywords**: [parent*]) *OR*(**Keywords**: [Caregiver]) *OR* (**Keywords**: [Care‐Giver]))) *OR* (((**abstract**: [Flexib*]) *OR* (**abstract**: [Resilien*]) *OR* (**abstract**: [Positiv*]) *OR* (**abstract**: [Enhanc*]) *OR*(**abstract**: [Develop*]) *OR* (**abstract**: [Increas*]) *OR* (**abstract**: [Improv*]) *OR*(**abstract**: [“Better”]) *OR* (**abstract**: [Strong*]) *OR* (**abstract**: [“Enhanced Maturity”]) *OR* (**abstract**: [“Increased Well‐Being”]) *OR* (**abstract**: [“Improved Interpersonal Relationships”]) *OR* (**abstract**: [“Improved Relationship”]) *OR* (**abstract**: [“Better Adjustment”]) *OR* (**abstract**: [“Improved Social Relationships”]) *OR* (**abstract**: [“Increased Self‐Efficacy”]) *OR* (**abstract**: [“Increased Independence”]) *OR*(**abstract**: [“Improved Identity”]) *OR* (**abstract**: [“Increased Acceptance”]) *OR*(**abstract**: [“Increased Autonomy”]) *OR* (**abstract**: [“Stronger Character”]) *OR*(**abstract**: [“Nurtured Spirituality”]) *OR* (**abstract**: [“Engaged Living”]) *OR* (**abstract**: [“Better Adjustment”]) *OR* (**abstract**: [“Life Satisfaction”]) *OR* (**abstract**: [“Valued Activities”]) *OR* (**abstract**: [parent*]) *OR* (**abstract**: [Caregiver]) *OR* (**abstract**: [Care‐Giver])))) *AND* ((((**Keywords**: [Flourish*]) *OR* (**Keywords**: [“Positive Change”]) *OR* (**Keywords**: [Grow*]) *OR* (**Keywords**: [Thriv*]) *OR* (**Keywords**: [Benefit]) *OR* (**Keywords**: [Goal]) *OR* (**Keywords**: [“Emotional Development”]) *OR*(**Keywords**: [Well‐Being]) *OR* (**Keywords**: [Well Being]) *OR* (**Keywords**: [Adjustment]) *OR* (**Keywords**: [Silver Lining]) *OR* (**Keywords**: [Silver‐Lining]) *OR*(**Keywords**: [Social]) *OR* (**Keywords**: [Change]) *OR* (**Keywords**: [Optimism]) *OR*(**Keywords**: [Hope]) *OR* (**Keywords**: [“Plans for Future”]) *OR* (**Keywords**: [Self‐Efficacy]) *OR* (**Keywords**: [Self Efficacy]) *OR* (**Keywords**: [“Sense of Control”]) *OR*(**Keywords**: [“Positive Affect”]) *OR* (**Keywords**: [Sustain*]) *OR* (**Keywords**: [Independence]) *OR* (**Keywords**: [Identity]) *OR* (**Keywords**: [“Developmentally Ahead”]) *OR* (**Keywords**: [Acceptance]) *OR* (**Keywords**: [Autonomy]) *OR*(**Keywords**: [Character]) *OR* (**Keywords**: [Spirit*]) *OR* (**Keywords**: [“Treatment of Others”]) *OR* (**Keywords**: [Engaged]) *OR* (**Keywords**: [“Purpose in Life”]) *OR*(**Keywords**: [Adjustment]) *OR* (**Keywords**: [Optimism]) *OR* (**Keywords**: [“Engagement in Meaningful Activities”]) *OR* (**Keywords**: [“Improved Quality of Life”]) *OR* (**Keywords**: [“Fulfilling Life”]) *OR* (**Keywords**: [Asset*]) *OR* (**Keywords**: (“Positive Mood”)) *OR* (**Keywords**: [“Post Traumatic Growth”]) *OR* (**Keywords**: (“Post‐Traumatic Growth”)) *OR* (**Keywords**: [“Adversarial Growth”]) *OR* (**Keywords**: [Satisf*]))) *OR* (((**abstract**: [Flourish*]) *OR* (**abstract**: [“Positive Change”]) *OR*(**abstract**: [Grow*]) *OR* (**abstract**: [Thriv*]) *OR* (**abstract**: [Benefit]) *OR* (**abstract**: [Goal]) *OR* (**abstract**: [“Emotional Development”]) *OR* (**abstract**: [Well‐Being]) *OR*(**abstract**: [Well Being]) *OR* (**abstract**: [Adjustment]) *OR* (**abstract**: [Silver Lining]) *OR* (**abstract**: [Silver‐Lining]) *OR* (**abstract**: [Social]) *OR* (**abstract**: [Change]) *OR*(**abstract**: [Optimism]) *OR* (**abstract**: [Hope]) *OR* (**abstract**: [“Plans for Future”]) *OR* (**abstract**: [Self‐Efficacy]) *OR* (**abstract**: [Self Efficacy]) *OR* (**abstract**: [“Sense of Control”]) *OR* (**abstract**: [“Positive Affect”]) *OR* (**abstract**: [Sustain*]) *OR*(**abstract**: [Independence]) *OR* (**abstract**: [Identity]) *OR* (**abstract**: [“Developmentally Ahead”]) *OR* (**abstract**: [Acceptance]) *OR* (**abstract**: [Autonomy]) *OR* (**abstract**: [Character]) *OR* (**abstract**: [Spirit*]) *OR* (**abstract**: [“Treatment of Others”]) *OR* (**abstract**: [Engaged]) *OR* (**abstract**: [“Purpose in Life”]) *OR* (**abstract**: [Adjustment]) *OR* (**abstract**: [Optimism]) *OR* (**abstract**: [“Engagement in Meaningful Activities”]) *OR* (**abstract**: [“Improved Quality of Life”]) *OR* (**abstract**: [“Fulfilling Life”]) *OR* (**abstract**: [Asset*]) *OR* (**abstract**: [“Positive Mood”]) *OR* (**abstract**: [“Post Traumatic Growth”]) *OR* (**abstract**: [“Post‐Traumatic Growth”]) *OR* (**abstract**: [“Adversarial Growth”]) *OR* (**abstract**: [Satisf*])))) *AND*((((**Keywords**: [“Chronic Pain”]) *OR* (**Keywords**: [Pain*]) *OR* (**Keywords**: [“Long‐Term Pain”]) *OR* (**Keywords**: [“Persistent Pain”]) *OR* (**Keywords**: [“Recurrent Pain”]))) *OR* (((**abstract**: [“Chronic Pain”])*OR* (**abstract**: [Pain*]) *OR* (**abstract**: [“Long‐Term Pain”]) *OR* (**abstract**: [“Persistent Pain”]) *OR* (**abstract**: [“Recurrent Pain”])))) *NOT* **Any Field**: Cancer

### 2.3 Study selection

Following completion of all database searches, references were imported into reference management software, Zotero (www.zotero.org), where duplicates were removed. Remaining references were imported into Covidence (www.covidence.co.uk), a specialized software used for managing review data for screening. After further duplicates were removed, all remaining papers were screened against title and abstract by the first author (RP). A total of 60% of papers were double screened by trained research assistants (CH, AD, AO), a process that has been followed in other research (^10^: 50% double screened). Any conflicts were resolved by co‐author AJ.

At the title and abstract screening stage, papers were included if they were related to adolescents aged 10–24 years old (congruent with a recent extended definition of adolescence of 10–24 years^30^) who live with chronic pain conditions as their primary concern, or parents of such adolescents, and addressed findings which explicitly related to flourishing in adolescents with chronic pain. For the purposes of our database search and study selection, we adopted a broad definition of flourishing in order to include as many papers as possible to enable us to best address our research questions. The definition used was as follows: ‘Flourishing in this review describes any positive changes as a result of living with chronic pain, beyond only an absence of negative outcomes. The positive aspect should not only be a result of an intervention or treatment but rather naturally occur due to the person's experience with chronic pain’. Eligible papers included those which adopted a qualitative, quantitative, or mixed method approach, were longitudinal, cross‐sectional or intervention/experimental (randomized controlled trials) studies. Additionally, eligible papers were required to be published primary research studies which reported on original findings in peer‐reviewed journal articles, conference abstracts, or unpublished theses. Papers were excluded if they were related to adolescents, or parents of such adolescents, who were not aged 10–24 years old, who only had pain which appeared to be a secondary concern, who had pain which was due to cancer (due to the potential life‐threatening aspect of cancer and the unique causes of pain associated with cancer, for example, tumor pressing on nerves or nerve damage due to cancer treatment), or if papers did not address findings which explicitly related to flourishing in adolescent chronic pain. Studies were also excluded if they were editorials, commentaries, case studies, review papers, letters, books, or book chapters. Papers were retained for further full‐text consideration if it was not possible to infer suitability for inclusion or exclusion based on title and abstract screening alone.

Following the title and abstract screening stage, remaining papers were assessed for full‐text eligibility. As guided by Arksey and O'Malley,^26^ these papers were all double screened by the first author (RP) and a fully trained research assistant (SG) using the same inclusion and exclusion criteria as at the title and abstract screening stage. Conflicts were resolved by co‐author, JM.

### 2.4 Charting the results

Data extraction for the included papers was conducted by the first author (RP) using a data extraction template devised by Peters, Godfrey, et al.^31^ Data extracted was also completed independently by a trained research assistant (AD) and compared with RP to ensure accuracy of the data extracted for each eligible study. According to this template, data were extracted for the following: author(s), year of publication, study aims/purpose/objective, origin/country of origin, participant characteristics and sample size if applicable (age, gender, racial, cultural), type of pain, duration of pain, methodology (e.g., self‐report, interview), study design (qualitative, quantitative, or mixed methods), and duration of the study. In addition, outcome data particularly relevant to the research questions were extracted for the following: key outcomes which relate to flourishing in adolescents with chronic pain, such as definitions, impact, assessment, gaps, and future research. Following data extraction, a narrative synthesis was conducted to analyze findings from the eligible papers, as part of the process of charting the data.^26^ This synthesis focused on generating codes which identified important features of the included papers. These codes were subsequently examined and collated to identify commonalities among the papers. Following this, commonalities were compared with the data to identify final patterns present across the papers, moving beyond merely a description of each individual study (such as the approach taken by Hynes et al.^32^). The data from the final four papers were further collated and reported in relation to the review questions, by examining the included papers to search for common findings and the occurrence of flourishing.

### 2.5 Quality assessment

To ensure rigor, data were subjected to quality assessment using a tool developed by Alderfer et al.^29^ This assessment was undertaken by the first author (RP) and a research assistant (AD) at the point of data extraction. A maximum of 16 quality criteria were used (quantitative methods studies rated on 9 criteria, qualitative methods studies rated on 11 criteria and mixed methods studies rated on 16 criteria), with a rating of between 1 (low quality, little to no evidence of guideline fulfillment) and 3 (high quality, good evidence of guideline fulfillment). Ratings were then averaged to obtain a total scientific merit score for each paper.

## 3 RESULTS

Following completion of all database searches, a total of 9086 references were imported in Zotero to screen for duplicates. Following this, 8814 remaining references were imported into Covidence for further screening. After another 1488 duplicates were removed, 7326 remaining papers were screened against title and abstract, resulting in 7318 papers being excluded and eight remaining papers being assessed for full‐text eligibility. Following full‐text screening, a further four papers were excluded, resulting in a final four papers^6^, ^33^, ^34^, ^35^ being included in the review.

A limited gray literature hand search of conference abstracts, unpublished theses and book chapter reference sections identified one potentially relevant book chapter reference, 17 potentially relevant conference abstracts and one potentially relevant result from an unpublished thesis. However, all of these were ultimately excluded as they did not meet the eligibility criteria. Reference sections of, and papers citing, the final four papers included in this review were also searched but produced no relevant results.

**FIGURE 1:**
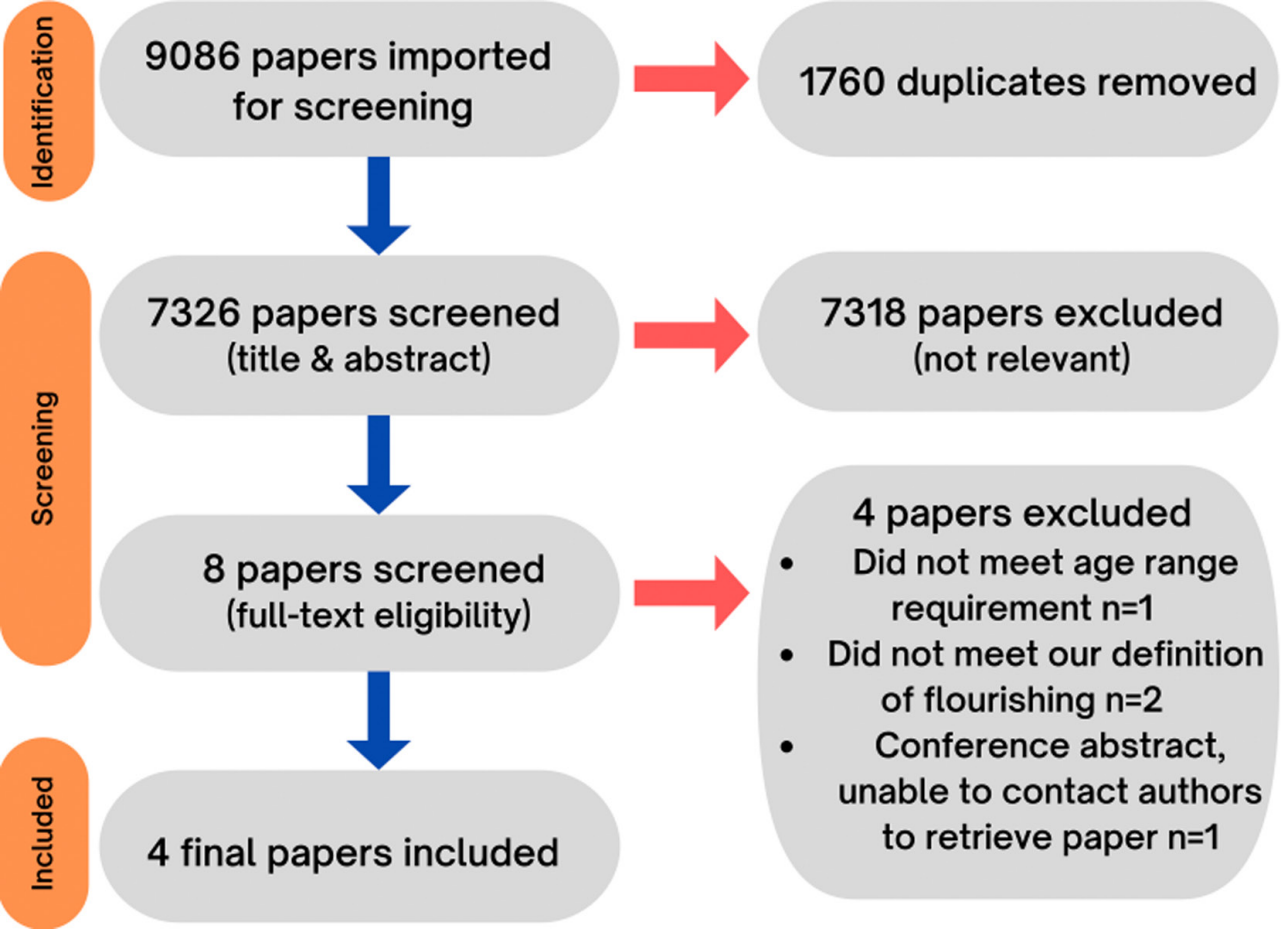
PRISMA flow diagram of screening process. Findings are now presented in relation to each of the three research questions.

No papers were excluded from the review based on the scientific merit quality rating (1–3), as all papers were rated highly on average by both reviewers, ranging from 2.18 to 2.55, *M* = 2.42 (*SD* = 0.12). The results of this scoping review are reported in accordance with Preferred Reporting Items for Systematic Reviews and Meta‐Analyses extension for Scoping Reviews (PRISMA‐ScR) guidelines.^28^ See Figure 1 for a PRISMA flow diagram of the screening process, including reasons for exclusion of the papers at full‐text screening stage.

### 3.1 Scope of the literature (research question 1)

The results of the review indicate that the scope of the current literature on flourishing in pediatric chronic pain is highly limited, with only four papers matching the search criteria. The papers were included in the scoping review based on their characteristics, which are summarized in Table 3.

**TABLE 3:** Overview of included papers' characteristics

Paper	Study Title	Aims of Study	Country	Sample Size	Mean Age	Gender	Racial Background	Study Design	Assessment Methodology	Type of Pain	Mean Duration of Pain (years)
Cartwright et al. (2015)	Journeys of adjustment: the experiences of adolescents living with juvenile idiopathic arthritis	To explore adolescents' experiences of living with JIA, with a particular focus on adjustment	United Kingdom	10	14.9	‐7 Female, ‐3 Male	White	Qualitative	Semi‐structured interviews	100% JIA	4.8
Eccleston et al. (2008)	Adolescent social development and chronic pain	To investigate the psychosocial impact of pain on social development	United Kingdom	110	15.1	‐80 Female, ‐30 Male	Not reported	Quantitative	Self‐report measures	‐47 Mulitple site idiopathic pain ‐41 CRPS ‐9 Chronic Headache ‐7 Low back pain ‐6 Recurrent abdominal pain	4.1
Jordan et al. (2016)	‘You have to be a jack of all trades’: Fathers parenting their adolescent with chronic pain	To examine the experiences of being a father of an adolescent with chronic pain	United Kingdom	6	15.1 (adolescents), 37.5 (fathers)	‐3 Female, ‐3 Male (adolescents), ‐6 Male (Fathers)	White	Qualitative	Semi‐structured interviews	‐4 Localized Idiopathic Pain ‐1 Diffuse Idiopathic Pain ‐1 Chronic Regional Pain Syndrome Type 1	>3 months (no mean reported)
Soltani et al. (2018)	Finding Silver Linings: A preliminary examination of benefit finding in youth with chronic pain	To examine the construct of benefit finding in youth with chronic pain	Canada	145	13.3	‐Approx. 98 Female, ‐Approx. 47 Male	‐81.3% White ‐7.6% Multi‐racial ‐4.2% Asian ‐3.5% Latin American ‐2.8% Other ‐0.7% No answer	Quantitative	Self‐report measures	‐61% Headache ‐38% Complex pain ‐1% Abdominal pain	2.95

Papers were published in 2008, 2015, 2016, and 2018, with *n* = 3 papers originating in the United Kingdom and *n* = 1 in Canada. Three papers focused on adolescents living with chronic pain, while one paper^34^ examined the experiences of fathers of adolescents with chronic pain. Sample sizes varied between papers, with the two qualitative papers recruiting sample sizes of *n* = 6 and *n* = 10, and the two quantitative papers recruiting sample sizes of *n* = 110 and *n* = 145. The mean age of participants in the three adolescent focused papers was *M* = 14.43 years, while the mean age for fathers reported in Jordan et al.^34^ was *M* = 37.5 years. Participants were predominantly female in all papers (70%), apart from the parent focused study (100% fathers). While three of the papers explored multiple types of chronic pain (including Complex Regional Pain Syndrome, idiopathic pain, chronic headache, and lower back pain), one paper^33^ focused solely on Juvenile Idiopathic Arthritis (JIA). With regard to study design, two papers adopted a qualitative design^33^, ^34^ while two papers adopted a quantitative design.^6^, ^35^ Both of the papers that adopted a qualitative design used semi‐structured interviews to generate qualitative data, investigating adjustment in adolescents living with JIA^33^ and father's experiences of parenting an adolescent living with chronic pain.^34^ The two quantitative papers used self‐report measures at a single time point to investigate the psychosocial impact pain may have on social development,^6^ and the relationships between benefit finding and internalizing mental health symptoms, quality of life, and pain‐related outcomes.^35^

### 3.2 Defining flourishing (research question 2)

To address the research question regarding the definition and understanding of flourishing in pediatric pain literature, commonalities were identified among the four papers. These commonalities may prove useful in supplementing our definition of flourishing in adolescent chronic pain and included: benefit finding, enhanced maturity and growth, and social support.

#### 3.2.1 Benefit finding

A dominant commonality within the eligible papers was benefit finding in the context of living with adolescent chronic pain. Benefit finding may be considered an important aspect of flourishing and is defined by Soltani et al.^35^ as the perception of positive consequences despite adversity.

All four studies observed benefit finding among participants. While Soltani et al.^35^ was primarily focused on benefit finding, the remaining three papers were primarily focused on other variables but observed aspects of benefit finding in line with the above definition. Three of the papers observed benefit finding among adolescents,^6^, ^33^, ^35^ while one paper observed parental (paternal) benefit finding.^34^ Benefit finding in two of the papers^6^, ^35^ was observed using quantitative self‐report measures such as the *Benefit and Burden Scale for Children* ^36^ used in Soltani et al.^35^ Conversely, benefit finding was reported in the remaining two papers^33^, ^34^ in qualitative interview data with adolescents and parents.

In the Soltani et al.^35^ paper, the researchers found that benefit finding was significantly positively associated with anxiety (*r* = 0.32, *p* = <0.001), depression (*r* = 0.29, *p* = 0.001), and Post‐Traumatic Stress Disorder (*r* = 0.30, *p* = 0.002). Benefit finding was also positively associated with higher pain intensity (*r* = 0.26, *p* = 0.002) and pain interference (*r* = 0.25, *p* = 0.002), indicating that higher levels of benefit finding may be associated with negative outcomes. In addition, benefit finding was found to be negatively associated with quality of life (*r* = −0.31, *p* = <0.001). Although some of these results may appear surprising, Soltani et al.^35^ offer the explanation that these results may reflect the complexity of chronic pain, and that past literature on chronic pain recognizes both highs (resilience) and lows (distress), which may change over the course of treatment.^37^

A common finding relating to benefit finding in adolescent chronic pain was identified among two of the papers^33^, ^34^ in this review. Namely, that individuals adopted a more positive outlook due to living with their chronic pain. In Cartwright et al.,^33^ this was demonstrated by adolescents living with JIA who reported benefits due to living with their chronic pain, such as developing a positive ‘can do’ attitude and feelings of gratitude regarding current and future functioning. These positive outcomes were linked to regaining a sense of control. Cartwright et al.^33^ describe how adolescents living with JIA reported being resilient to negative feelings and thoughts, which appeared to be facilitated by regaining a broader sense of control over their body and life. Specific strategies for regaining control were identified by adolescents, including taking charge and overcoming limitations (leading to positive attitudes), downward comparisons with those more disabled (leading to positive emotions), and the choice of disclosure of their condition to others which was something which they could control. Of relevance in this paper was how participants altered their outlook and behavior to be more positive, which in turn appeared to lead to positive outcomes and benefit finding. For example, qualitative findings included how living with JIA had enabled the discovery that maintaining positivity leads to things going in one's favor. All participants discussed the importance of altering their outlook to having a positive attitude and acceptance of the condition. Similarly, a focus was placed on positive change in Jordan et al.^34^ Fathers in this study reported learning to reappraise what is important to them about their relationship with their child and learning to focus on the child's current quality of life rather than on shared activities that were no longer possible. This led to a strengthening of their relationship through experiencing shared adversity together.

#### 3.2.2 Enhanced maturity and growth

A second common finding among two of the papers^6^, ^33^ was enhanced maturity and growth. Adolescents in Cartwright et al.^33^ reported self‐growth and reappraisal in life due to the challenges they experienced as a result of living with JIA. Such experiences were described as character forming, resulting in the development of strength as an individual and positive trait. Qualitative findings also showed that participants reported adopting a more extroverted personality in social situations to enable them to cope with their pain condition, resulting in positive social outcomes. Similarly, to Cartwright et al.,^33^ enhanced maturity was also reported by adolescents in relation to living with chronic pain in the quantitative paper of Eccleston et al.^6^ This was demonstrated in adolescents' perceptions of their superior ability to solve problems when compared to their peers, which can be considered a developmental advancement. It is important to note, however, that participants rated themselves developmentally ahead of their peers in only this one item out of 11 items measured, while participants rated themselves as either developmentally similar to (five items) or behind (five items) their peers for the remaining items in this study.

#### 3.2.3 Social support

Social support was identified as a commonality among three of the papers.^6^, ^33^, ^34^ In Cartwright et al.,^33^ adolescents living with JIA reported gaining additional social support from friends when choosing to disclose their condition. Greater awareness of love and support from family as well as closer friendships were also mentioned as positive outcomes by adolescents, which developed from living with their pain condition and was identified by the authors as an important coping resource. Family and friends provided a sense of normality and hope, a possible contributor to flourishing. In Jordan et al.,^34^ fathers of adolescents living with chronic pain reported a positive change in a strengthened relationship with their adolescents, particularly in relation to engaging in shared activities. The main positive outcome noted in Eccleston et al.^6^ of maturity in dealing with problems was likewise attributed to the adolescent's experience of coping, which was linked to a social aspect with peers. Three factors of social development were self‐perceived in adolescents with chronic pain; emotional adjustment (made up of three items associated with adolescent's behavior regarding competence and emotional problem solving), identity formation (made up of four items associated with adolescent's socially assured behavior), and independence (made up of four items associated with adolescent's behavior independent of parents). Across these three factors, strong peer relations appeared to play a protective role and were associated with positive judgments of the adolescent's social development.^6^

### 3.3 Gaps in the literature and directions for future research (research question 3)

Numerous gaps in knowledge were identified when collating these results. Most importantly, only four papers were identified which matched the search criteria for flourishing in adolescent chronic pain. In addition, only two of these papers specifically focused on potential contributors to flourishing (^33^: adjustment and adaptation to living with JIA,^35^: benefit finding), while the remaining two papers were primarily focused on other areas (^6^: social development of adolescent with chronic pain,^34^: parenting an adolescent with chronic pain), with flourishing only included in the findings as part of their larger results. Furthermore, no papers offered suggestions for a general definition of flourishing. In addition to the overall highly limited literature identified, review of the papers highlighted a lack of diversity of participants in relation to gender, racial, ethnic, and cultural backgrounds, and a reliance on cross‐sectional, self‐report or interview methodology. Cartwright et al.^33^ note the limitations of their small sample of predominantly white participants recruited from a single clinical center, as well as data collection at single time point. Similarly, Soltani et al.^35^ note the limitations of their participants being primarily white middle‐class and the need for longitudinal research. Eccleston et al.^6^ and Jordan et al.^34^ also note the absence of, and need for, a longitudinal approach to research on the effect of pain on development, and parenting an adolescent with chronic pain, respectively.

## 4 DISCUSSION

The aim of this scoping review was to provide an overview of the current scope of the literature, document how flourishing is defined and understood in the literature and identify the current knowledge gaps to direct future research. A total of four eligible papers were identified with commonalities of benefit finding, enhanced maturity and growth, and social support identified within the findings. Notably, this review identified a dearth of literature and knowledge regarding what is known about how flourishing is defined and understood in the context of adolescent chronic pain, and how it is studied.

To provide further clarity on a definition of flourishing in chronic pain, we made use of constructs identified in our included papers to identify commonalities related to flourishing among these studies. Findings from our review support the inclusion of benefit finding, enhanced maturity and growth, and social support in defining flourishing in adolescents living with chronic pain. Thus, flourishing may be seen as either a predictor of positive outcomes or as a positive outcome in itself. For example, the construct of benefit finding, which we believe to fall under the umbrella term of flourishing, may predict positive outcomes such as growth, while growth as a positive outcome may be seen as flourishing in itself. These findings tie in with previous suggested definitions of flourishing, including psychological, social, and emotional well‐being^15^, ^16^ and sustainability despite adversity^8^ with a strong emphasis on positive outcomes or change, and growth. Benefit finding was identified as a major commonality present across all papers and a likely key contributor to flourishing in adolescent chronic pain. This was demonstrated in the way that participants focused on the benefits of their current abilities despite living with their chronic pain and associated negative outcomes. Behaviors described by participants such as adopting a more positive outlook and developing a more outgoing personality leading to more positive outcomes, are congruent with the Broaden and Build Theory^38^ which forms part of the Positive Psychology tradition. This theory postulates that positive emotions may be used to broaden a person's attention and awareness towards positive aspects which may still be present in their lives.^8^ In the long term, this broadened perspective can then lead to a building of personal resources, including physical, psychological, and social resources such as increased social support.^38^

Since a combination of biological and psychosocial variables may influence pain in each individual,^39^ it is important to consider flourishing within the broader social context in which an adolescent's pain is experienced. Findings from the included studies indicate that social support from family and peers may have a positive effect on pain outcomes and provide additional resources to promote positive behaviors in adolescents living with chronic pain. Likewise, relationships between parents and their child may be strengthened due to adolescent chronic pain,^34^ a positive outcome for both family members. Additionally, strong social relationships may be altered to mitigate the negative effects of pain,^20^ leading to positive changes. Further resources may be provided by parents who promote coping, pain management and adjustment in their child^40^ to encourage these adaptive behaviors. Social support from family and friends can also lead to perceived positive outcomes such as a greater awareness of love and support, as well as the direct benefit of strengthened friendships. In addition, this support may provide a coping resource and lead to hope, a concept linked to flourishing.^41^ As indicated in one of the papers included in this review, strong friendships may also lead to perceived strengthened social development, and strong relationships with peers have been linked to positive emotional adjustment and independence in adolescents living with chronic pain.^6^

The link found in this review between social support and positive outcomes, including growth, is further emphasized in the broader literature on Post‐Traumatic Growth. Meyerson et al.^42^ conducted a comprehensive systematic review on Post‐Traumatic Growth in young people and found preliminary support for the link between Post‐Traumatic Growth and social support. In a more pain specific study by Dirik and Karanci^19^ in adults living with Rheumatoid Arthritis, perceived social support appeared to be a significant predictor of Post‐Traumatic Growth.

Importantly, gaps identifying research needs, limitations, and directions for future research were identified among the four papers: Firstly, the majority of participants included in papers in this review were of a White racial background. In addition, three of the four papers were conducted in the United Kingdom, with only one study conducted in Canada. Likewise, our review only included studies which were published in English. This focus on exclusively Western countries highlights the issue of research studies focusing primarily on participants from Western Educated Industrialized Rich Democratic (WEIRD) societies for data collection, which are not representative of the global population.^43^ Further studies are therefore needed to be fully representative and thoroughly investigate the occurrence of flourishing in adolescent chronic pain across multiple contexts. Secondly, most adolescents were female. Although rates of chronic pain are typically higher in female adolescents,^44^ further studies involving male participants would be useful to investigate whether there may be differences in results due to sex and gender differences. Likewise, in the only study involving parents, all parent participants were male. Further studies involving flourishing in mothers of adolescents living with chronic pain would be useful to investigate their experience of flourishing and how this may differ from those identified by fathers in Jordan et al.^34^ Thirdly, as we have noted, social factors in general appear to play an important role in contributing to flourishing in adolescent chronic pain. However, social considerations of family and peers were only included in two of the papers. While there may be a temptation to focus solely on adolescents living with chronic pain themselves in future studies, the potential importance of familial factors on adolescent flourishing warrants further investigation. In addition, very little has been investigated on the important role peers may play in those living with chronic pain.^6^ Future studies should include a consideration of an adolescent's wider social circle, to take account of the wider social context in adolescent chronic pain. Fourthly, all papers in this review used either questionnaires or interviews as their methodology. Despite this, there are insufficient developmentally appropriate and psychometrically robust measures available to sufficiently assess flourishing as well as adaptive functioning in the context of adolescent chronic pain, which may possibly explain the dearth of studies in this area. It may also be fruitful to qualitatively examine what flourishing means to adolescents themselves and their perceptions on how this may be pursued. Studies using a variety of other methodologies could usefully provide a more detailed exploration of flourishing in the context of adolescent chronic pain, by developing our understanding of everyday experiences and subjectivity in chronic pain. In addition to interview studies, diary studies may be used as a way of capturing details of everyday experiences related to chronic pain, while avoiding recall issues with methods that require retrospective recall.^45^ Another methodological approach which may be considered is Q‐methodology. Q‐methodology is a robust methodology and could be used to further explore subjectivity in relation to flourishing in adolescent chronic pain by investigating dominant shared viewpoints across multiple stake holders associated with adolescent chronic pain (e.g., the adolescent, their parents, their peers, and healthcare professionals who are involved in their care and treatment). There is also a lack of longitudinal studies in the literature, which would be useful to examine the construct of flourishing over time and its predictors. Additionally, it may also be useful to investigate flourishing in particular conditions, as there may be differences in levels of flourishing experienced based on different causes of chronic pain and their related severity and intensity. Finally, we note the potential important influence co‐occurring mental health symptoms may have on adolescent chronic pain.^46^ These symptoms may influence the relationship between flourishing and chronic pain, as adolescents with chronic have been shown to be twice as likely to report high emotional distress than those not living with chronic pain.^5^ Future research should consider flourishing in adolescents living with co‐occurring chronic pain and diagnosable mental health conditions, and the specific nature of the relationship between flourishing and mental health factors.

There were also some limitations in our review. Firstly, our search only included papers published in the English language. Due to a lack of capacity to translate papers in other languages, it is possible that relevant results to our searches may have been missed or overlooked. Secondly, we were unable to retrieve the full paper (if written) for a potentially relevant conference abstract which was identified as potentially eligible for inclusion in the review.

Further studies examining flourishing could be used to explore and build on this overlooked but potentially valuable resource in supporting adolescents living with chronic pain. Results from such studies could be useful to inform treatment strategies based on Positive Psychology for adolescents with chronic pain and their parents, which draw on existing strengths and capabilities to support the self‐management of chronic pain among adolescents. Positive Psychology interventions have been shown to be beneficial for adults living with chronic pain^47^, ^48^ and successful positive interventions have been effective in other related areas such as psychological flexibility and acceptance.^18^, ^49^ This research could be further developed or drawn upon, to support adolescents living with chronic pain in self‐management of their pain symptoms.

In conclusion, this review, although based on a limited number of studies, suggests that there is a lack of research into flourishing among adolescents living with chronic pain and parents of such adolescents. Despite this, it is clear from the four papers identified in this review that adolescents living with chronic pain can indeed experience positive outcomes along with the predominant negative outcomes usually associated with living with chronic pain. This warrants further investigation to further explore flourishing, not only for the individual adolescent, but also for their wider social environment.

## FUNDING INFORMATION

This study was undertaken as part of a PhD Studentship funded by the Pain Relief Foundation.

## CONFLICT OF INTEREST

None of the authors have any conflicts of interest to declare.
